# Recurrent renal secondary hyperparathyroidism caused by supernumerary mediastinal parathyroid gland and parathyromatosis: A case report

**DOI:** 10.3389/fsurg.2023.1135596

**Published:** 2023-03-20

**Authors:** Longfei Li, Chenchen He, Guangming Cheng, Junying Cao, Chunhui Wang, Yufu Tang, Wei Zhang

**Affiliations:** ^1^ Department of Hepatobiliary and Thyroid Surgery, General Hospital of Northern Theater Command, Shenyang, China; ^2^ Department of Clinical Medicine and Surgery, China Medical University, Shenyang, China

**Keywords:** recurrence, parathyromatosis, supernumerary parathyroid glands, hyperparathyroidism, secondary, case reports

## Abstract

**Background:**

Surgical parathyroidectomy (PTX) is necessary for patients with severe and progressive secondary hyperparathyroidism (SHPT) refractory to medical treatment. Recurrence of SHPT after PTX is a serious clinical problem. Both supernumerary mediastinal parathyroid gland and parathyromatosis are the rare causes of recurrent renal SHPT. We report a rare case of recurrent renal SHPT due to supernumerary mediastinal parathyroid gland and parathyromatosis.

**Case presentation:**

A 53-year-old man underwent total parathyroidectomy with autotransplantation due to the drug-refractory SHPT 17 years ago. In the last 11 months, the patient experienced symptoms including bone pain and skin itch, and the serum intact parathyroid hormone (iPTH) level elevated to 1,587 pg/ml. Ultrasound detected two hypoechoic lesions located at the dorsal area of right lobe of the thyroid gland, and both lesions presented as characteristics of hyperparathyroidism in contrast-enhanced ultrasound. ^99m^Tc-MIBI/SPECT detected a nodule in the mediastinum. A reoperation involved a cervicotomy for excising parathyromatosis lesions and the surrounding tissue and a thoracoscopic surgery for resecting a mediastinal parathyroid gland. According to a histological examination, two lesions behind the right thyroid lobe and one lesion in the central region had been defined as parathyromatosis. A nodule in the mediastinum was consistent with hyperplastic parathyroid. The patient remained well for 10 months with alleviated symptoms and stabilized iPTH levels in the range of 123–201 pg/ml.

**Conclusion:**

Although rare, recurrent SHPT may be caused by a coexistence of both supernumerary parathyroid glands and parathyromatosis, which should receive more attention. The combination of imaging modalities is important for reoperative locations of parathyroid lesions. To successfully treat parathyromatosis, all the lesions and the surrounding tissue must be excised. Thoracoscopic surgery is a reliable and safe approach for the resection of ectopic mediastinal parathyroid glands.

## Introduction

Secondary hyperparathyroidism (SHPT) is a common complication of end-stage renal disease (ESRD) (1). SHPT can causes bone pain and itching (2, 3), which contribute to the poor quality of life in dialysis patients. Moreover, prolonged SHPT is significantly associated with high incidences of cardiovascular events and all-cause mortality (4, 5). Surgical parathyroidectomy (PTX) is necessary for patients with severe and progressive SHPT refractory to medical treatment (6). A mountain of evidence identified that PTX can alleviate clinical symptoms, improve the quality of life, and reduce the risk of all-cause and cardiovascular mortality in patients with severe and progressive SHPT (7–9). Although PTX is generally a successful treatment for SHPT patients subjected to surgery, a significant proportion develop recurrent SHPT after PTX, ranging between 5% and 38%. Recurrent SHPT is mainly caused by the hyperplastic autografted tissue, remnant parathyroid tissues left in the neck, and the presence of ectopic and/or supernumerary parathyroid glands (SPGs) (10–14). SPGs are found commonly in the thymus, and the presence of SPGs in the mediastinum is rare in patients with SHPT (11, 12, 14). Moreover, parathyromatosis is a very rare cause of recurrent SHPT, which is characterized by hyperfunctional parathyroid foci in the parathyroid autotransplant sites and the primary surgery site (15, 16). There is a lack of discussion on the optimal management for recurrent SHPT, caused by ectopic mediastinal parathyroid glands or parathyromatosis, in the literature. Herein, we present an unusual case caused by mediastinal supernumerary parathyroid glands and parathyromatosis.

## Case presentation

A 53-year-old male was admitted to our hospital in November 2021 with progressive aggravation of recurrent bone pain and skin itch for 11 months. He had a history of chronic glomerulonephritis for 27 years and hemodialysis for 25 years. Seventeen years ago, he was diagnosed with SHPT due to complaints of bone pain, skin itch, and a significant increase in serum intact parathyroid hormone (iPTH) (>1,000 pg/ml), and a surgical parathyroidectomy was required. During the initial operation, four glands were removed and then confirmed as hyperplasia histopathologically, and about 30 mg tissues from the smallest one among the resected glands were implanted into right forearm at the same time. The serum iPTH decreased to 85 pg/ml on postoperative day 1, which further indicated a successful surgery had been performed. After the initial operation, the patient did not receive any drug, and serum iPTH levels were stabilized in the range of 80.5–150 pg/ml during the 3-year follow-up. The serum iPTH levels increased to 400–500 pg/ml 14 years ago, and the cause of elevated iPTH level has been considered as hyperplastic autografts in other institutions. During this period, he did not receive any imaging examinations. Then, the patient started to receive active vitamin D supplements of Rocaltrol treatment, and the iPTH levels were stabilized in the 150–300 pg/ml range until December 2020.

On December 2020, the patient visited our institution due to the return of symptoms including bone pain and skin itch. Routine laboratory tests showed that serum iPTH increased to 1,587 pg/ml, and calcium and phosphate levels were 2.38 mmol/L and 2.79 mmol/L, respectively. Recurrent renal SHPT was diagnosed, and the further examinations were recommended to locate the hyperfunctional parathyroid tissues, but the patient refused and medication was adopted. Unfortunately, therapies such as low phosphorus diet, phosphorus binders, calcitriol, calcium supplements, and vitamin D were ineffective.

**Figure 1:**
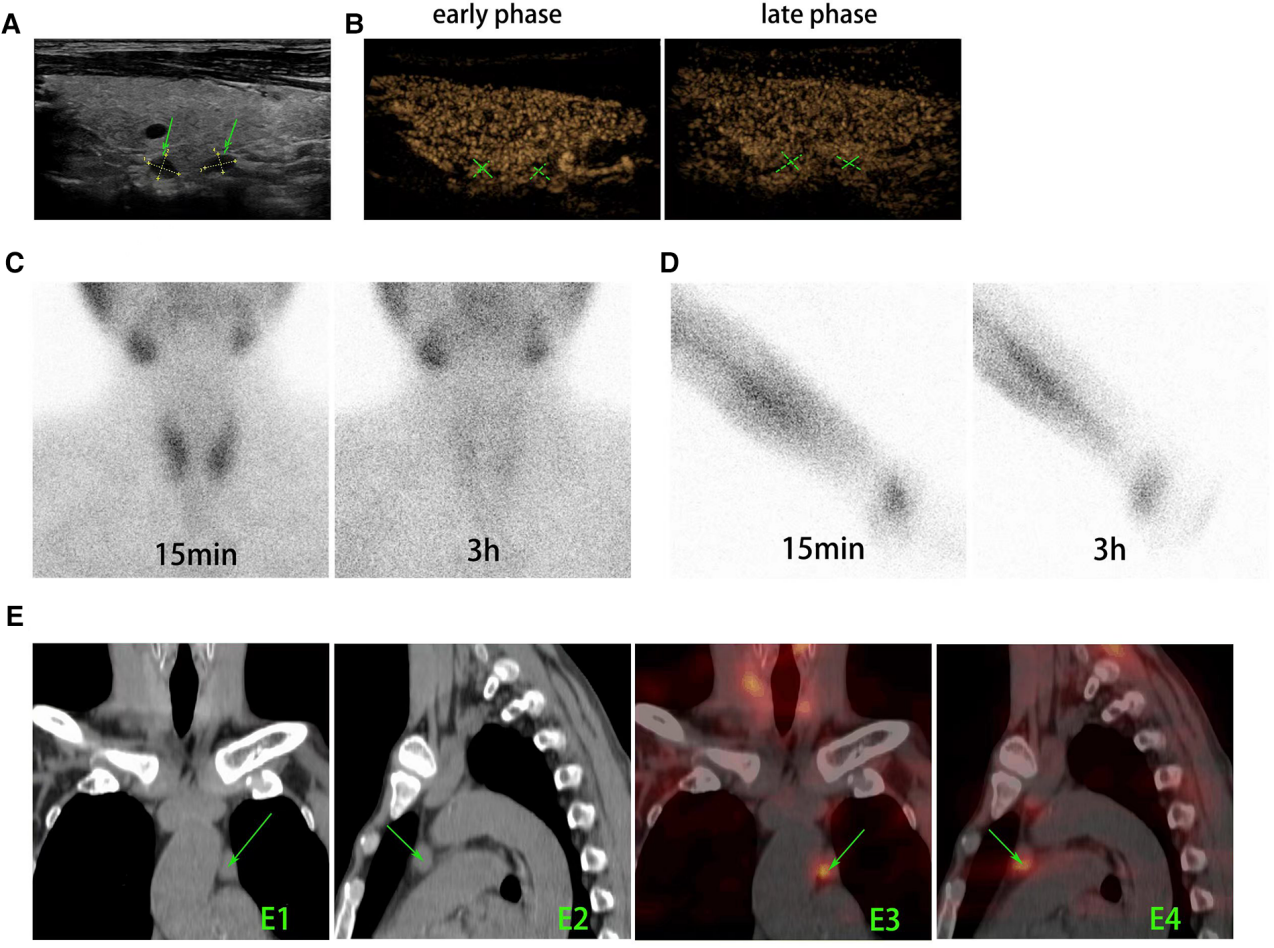
Preoperative imaging modalities were used to find and locate the parathyroid tissues. (**A**) US revealed two hypoechoic lesions with volumes of 0.55 cm × 0.45 cm and 0.55 cm × 0.35 cm (arrow) located at the dorsal area of right lobe of thyroid gland. (**B**) In CEUS, both lesions appeared as characteristics of parathyroid tissues (fast in the early phase, slow out in the late phase, and higher enhancement with a long enhancement time). (**C**) ^99m^Tc-MIBI planar early (15 min) and delayed (3 h) static images showed that there is no tissue with focal tracer accumulation presented at the primary surgery site of neck. (**D**) ^99m^Tc-MIBI planar early (15 min) and delayed (3 h) static images showed that there is no focal tracer accumulation at the autograft site. (**E**) The axial CT (**E1** and **E2**) and SPECT/CT (**E3** and **E4**) images showed soft-tissue masses with marked MIBI uptake in the left upper mediastinum (arrows). US, ultrasound; CEUS, contrast-enhanced ultrasound; ^99m^Tc-MIBI, ^99m^Tc-sestamibi.

**Figure 2:**
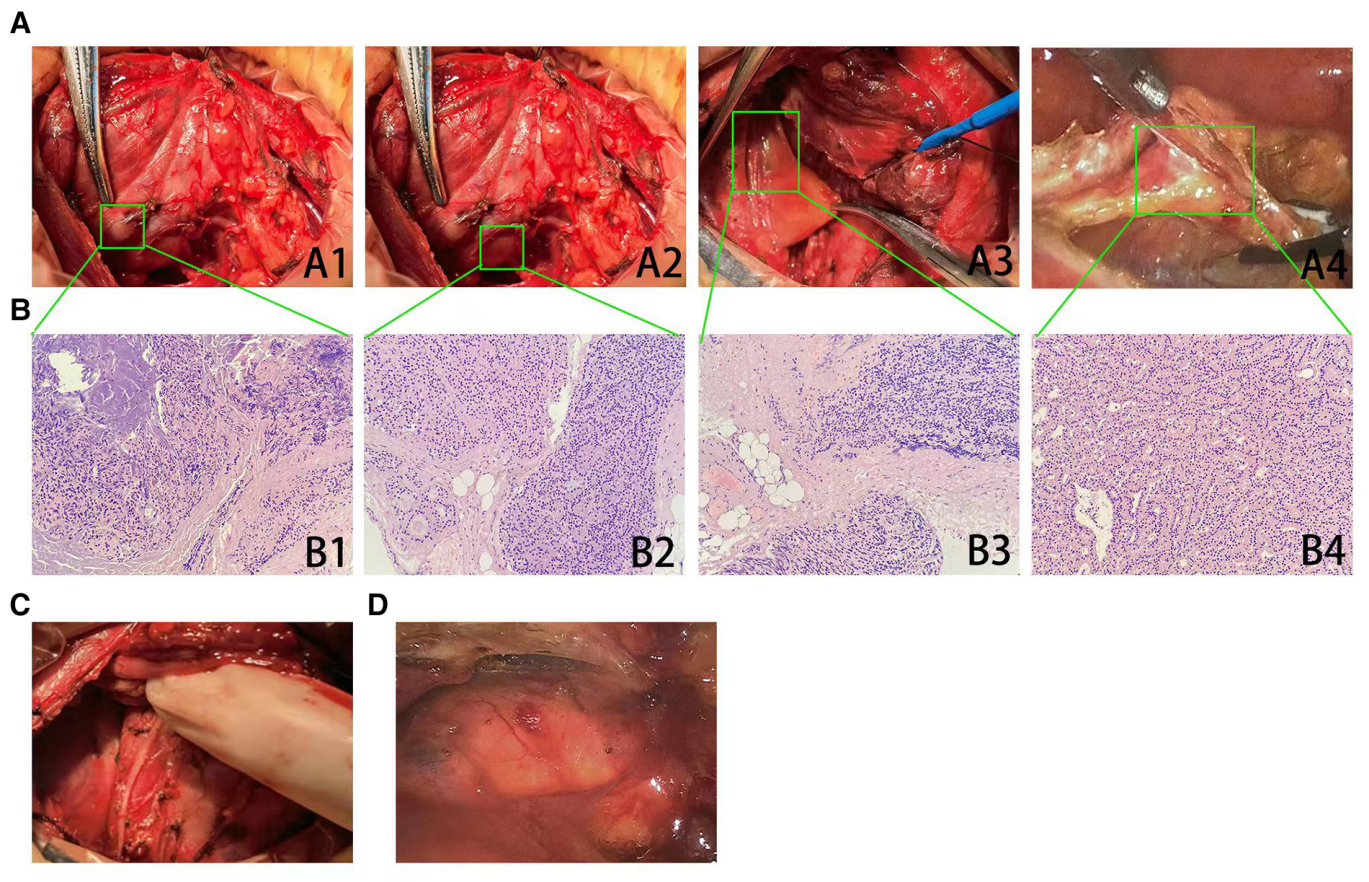
Excision of suspected recurrent parathyroid tissues and pathologic analysis. (**A**) Two suspected parathyroid lesions (**A**1–2, arrow) located at the dorsal area of right lobe of thyroid gland were resected. One suspected parathyroid lesion (**A**3, arrow) located on the central region of right neck was found during exploration, and then clearance of the central neck compartment was performed. A thoracoscopic surgery also was performed to remove a mediastinal parathyroid gland (**A**4, arrow). (**B**) Histopathology (hematoxylin–eosin staining  ×200) showed that the lesions at the site of primary surgery (**B**1–3) were consistent with the pathological features of parathyromatosis. The resected mediastinal parathyroid gland had demonstrated nodular hyperplasia of parathyroid tissue, which was well differentiated (**B**4). (**C**) An extensive surgery that involved the excision of suspected parathyroid lesions and the clearance of the central neck compartment was performed, and the postoperative picture had been shown. (**D**) A thoracoscopic surgery to remove mediastinal parathyroid gland had been performed, and the postoperative picture had been shown.

**Figure 3:**
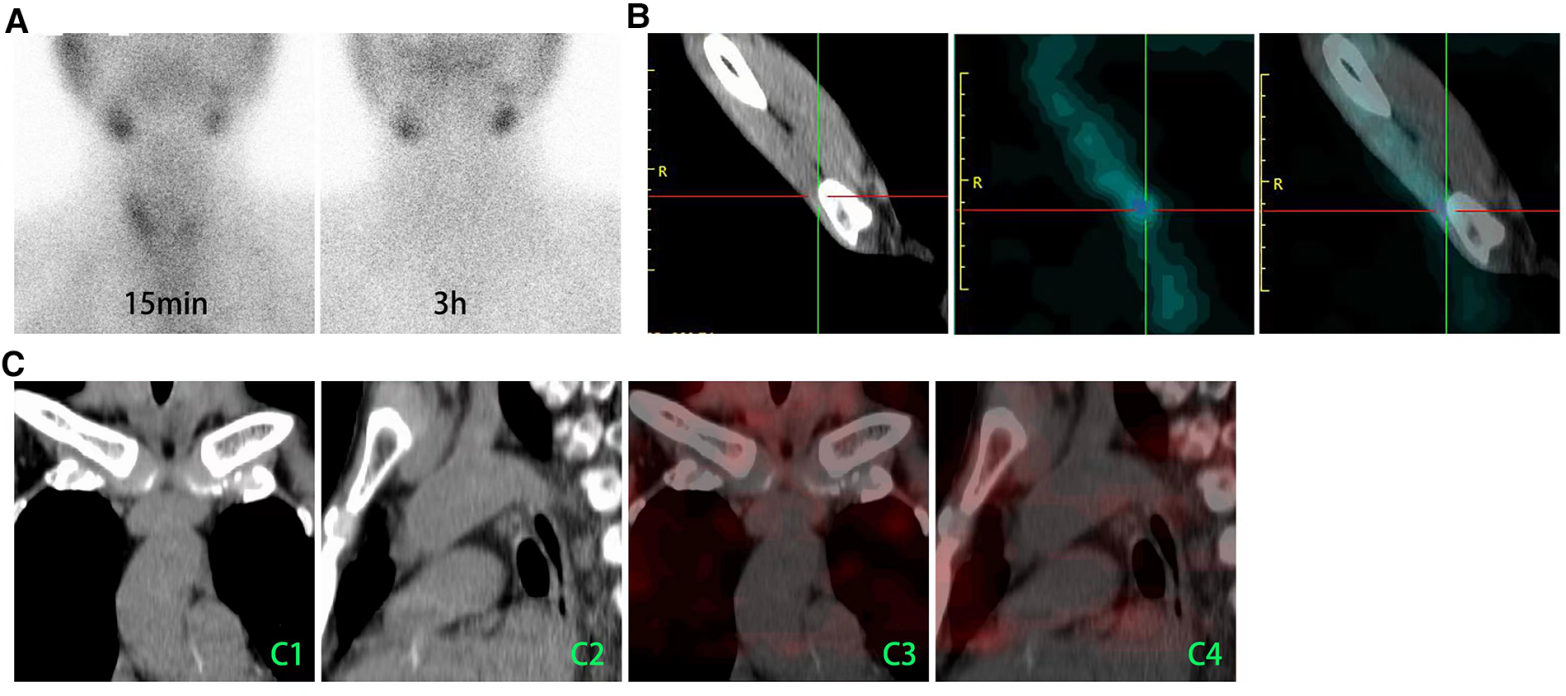
^99m^Tc-MIBI/SPECT was used to demonstrate whether the suspected parathyroid tissues had been missed. (**A**) ^99m^Tc-MIBI planar early (15 min) and delayed (3 h) static images showed that there is no tissue with focal tracer accumulation that presented in the primary surgery site of neck. (**B**) A weak focal tracer accumulation had presented in the autograft site. (**C**) The axial CT (**C**1 and **C**22) and SPECT/CT (**C**3 and **C**4) images showed no tissue with MIBI uptake in the mediastinum. ^99m^Tc-MIBI, ^99m^Tc-sestamibi; CT, computed tomography.

On November 2021, the patient visited our institution for evaluation again because of insupportable symptoms. Laboratory tests revealed that the serum iPTH, calcium, and phosphate were 1,100 pg/ml, 2.26 mmol/L, and 2.85 mmol/L, respectively. In the dorsal area of right thyroid lobe, ultrasound (US) detected two hypoechoic formations with volumes of 0.55 cm × 0.45 cm and 0.55 cm × 0.35 cm (Figure 1A), which presented as a vascularization pattern of parathyroid lesions (fast-in, slow-out, and higher-enhancement with long enhancement time) when examined by using contrast-enhanced ultrasound (CEUS) (Figure 1B). ^99m^Tc-sestamibi (^99m^Tc-MIBI)/single photon emission computed tomography (SPECT) did not find any tracer accumulation in the sites of neck and autograft but revealed a nodule (1.5 cm × 1.0 cm) with tracer uptake in the mediastinum (Figure 1C). Reoperation involved a cervicotomy and a thoracoscopic surgery was performed on 24 November 2021. Cervicotomy was performed to remove the suspected recurrent parathyroid tissue and the surrounding tissue around the right thyroid lobe (Figure 2A1–3). Besides two lesions of the neck detected by US and CEUS, another lesion was found in the central compartment of the neck during the reoperation (Figure 2A3), which made us believe that the hyperplastic parathyroid tissues at this region mainly resulted from a secondary implantation into the surrounding tissues of the damaged parathyroid gland during the initial operation. Therefore, the clearance of the central neck compartment also was performed (Figure 2C). A thoracoscopic surgery was performed to remove the hypertrophic mediastinal parathyroid gland (Figure 2A4). During surgery, an adequate PTH drop was achieved (246 pg/ml). Therefore, we did not perform a further exploration to make sure whether there are functional parathyroid tissues in the autografted site. According to the histological study, three lesions of parathyroid tissues at the site of primary surgery are represented by diffuse-nodular hyperplasia from dark main cells and nodular hyperplasia shown in fibroadipose tissues, and these lesions do not have their own capsule and suture material surrounded by nodular hyperplasia (Figure 2B1–3). Pathologic analysis of the resected mediastinal parathyroid gland showed nodular hyperplasia of parathyroid tissue, which was well differentiated (Figure 2B4). On the first day after operation, the iPTH level in a blood specimen obtained from the right arm was 185 pg/ml, whereas it was 123 pg/ml from the left arm. On the fifth day after operation, ^99m^Tc-MIBI/SPECT was performed, and no suspected parathyroid tissues were detected in the neck or the mediastinum (Figures 3A,C). Interestingly, a weak focal tracer accumulation had presented in the autograft site (Figure 3B), indicating the existence of functional parathyroid tissues in the autograft site. During 10 months of follow-up, his itching and bone pain significantly improved, and he had appropriate calcium (2.09–2.27 mmol/L) and iPTH (108–195 pg/ml, blood specimen from the left arm) levels under regular hemodialysis (Table 1).

**Table 1:** Summary of the case.

Background	Presentation	Evaluation	Surgical findings	Treatment	Outcome
53-year-old male, TPTX + AT due to the SHPT 17 years ago	Bone pain, skin itch, and elevated level of iPTH	Blood test, US, CEUS, and ^99m^Tc-MIBI/SPECT	Parathyroid gland in central compartment of the neck and mediastinum	Excision of parathyroid glands in the neck and mediastinum	Hospital discharge a week after surgery

TPTX + AT, total parathyroidectomy with autotransplantation; SHPT, secondary hyperparathyroidism; iPTH, intact parathyroid hormone; US, ultrasound; CEUS, contrast-enhanced ultrasound.

## Discussion

Recurrent SHPT is an uncommon yet challenging clinical problem (10, 17–22). Great efforts had been made to reduce the incidence of recurrent SHPT over the past decade. For example, previous studies had been performed to evaluate the recurrence of all types of surgical procedures (10, 17–20), and the results showed that total parathyroidectomy (TPTX) is superior with regard to the prevention of recurrence. However, TPTX can result in intractable postoperative hypocalcemia, a common complication that can increase mortality and hospitalization, and implementing long-term oral calcium and vitamin D is need after surgery (23). Considering the life-threatening sequelae, TPTX alone is not frequently performed for patients. Total parathyroidectomy with autotransplantation (TPTX + AT) has the advantage of avoiding long-term hypocalcemia but has an increased risk of recurrent SHPT induced by the autotransplanted parathyroid tissues (19, 22, 24).

Recurrent SHPT is one of the significant reasons for the dependence of transplant recipients on parathyroidectomy. Over time, the recurrence rate gradually increases and can reach up to 30% (24). First, this may be related to the pathological type of the transplanted parathyroid gland, as early parathyroid glands exhibit diffuse polyclonal proliferation, and late parathyroid glands may develop into adenomatous tissues due to certain genetic abnormalities and undergo monoclonal expansion (25). Second, in chronic kidney disease (CKD) patients who require dialysis, various physiological stimuli persist, including hyperphosphatemia, hypocalcemia, and lack of calcitriol. Under these circumstances, the parathyroid gland is more susceptible to hypertrophy, and both free and settled parathyroid cells eventually develop into glands with strong PTH secretion capacity (26). Additionally, the use of certain drugs may also promote the recurrence of secondary hyperparathyroidism (27).

SPGs have been demonstrated as a major cause of persistent or recurrent SHPT (11–13). Previous studies reported that SPGs had been found in 15%–39% of reoperative patients (12, 28). SPGs may result from separation of the parathyroid anlage during embryologic migration. SPGs usually present as parathyroid foci that cannot be detected by the recent imaging modalities and easily missed during the initial surgery (13, 29). These missed parathyroid foci, under ongoing stimulus of renal failure, would grow slowly and develop into the obvious glands and then the recurrence appears. In this case, the patient did have a long period of good recovery after the initial surgery, and an obvious gland had been found in the mediastinum that was determined as SPG during the reoperation. SPGs are found commonly in the thymus region, and a routine cervical thymectomy is recommended during the surgical treatment of patients with SHPT (11, 12, 14). Uno et al., for instance, in a retrospective study of 902 patients submitted to PTX and cervical thymectomy due to SHPT, found SPGs presented in 140 (15.5%) patients, located in the thymus region in 129 patients (92.1% of the number of patients with SPGs) (14). Importantly, previous studies demonstrated that the risk of recurrent SHPT in patients who underwent PTX and thymectomy is much lower than that in patients without thymectomy (12). Moreover, SPGs are also found to be located in the retroesophageal groove, carotid sheath, and mediastinum. It needs to be noticed that mediastinal localization of SPGs was initially low but its presence is not infrequent in the reoperative cases (12, 13). During reoperations, the incidence of mediastinal SPGs was estimated to be present in about 3.5%–30% of patients (12, 30). SPG excision here may necessitate sternotomy or thoracoscopy (31–34). In this case, we performed a thoracoscopic surgery to remove the mediastinal parathyroid gland. In accordance with previous studies (33, 34), we found the thoracoscopic approach for mediastinal SPGs to be feasible and safe.

Parathyromatosis is a rare cause of recurrent SHPT (15). There are two theories to explain the pathogenesis of parathyromatosis. The first (type 1) defines as preexisting parathyroid rests of embryological origin that undergo hyperplasia under the influence of physiological stimuli. The second (type 2), also known as the main cause of parathyromatosis, is a consequence of implantation into the surrounding tissues of the damaged parathyroid gland during the primary operation. In this case, one of the causes of recurrent SHPT was considered to be type 2 parathyromatosis because small and numerous nodules had been found in the primary surgical site and these nodules are surrounded by dense fibrous tissues. Optimal treatment for SHPT can be achieved by surgical resection, whereas in parathyromatosis, it is difficult to find and remove all the lesions intraoperatively hence requiring multiple surgeries. A previous literature study reported that only 4 of 10 patients were successfully diagnosed with parathyromatosis preoperatively, and the failure reoperation has been found in 6 of 10 patients with parathyromatosis (16). US may be more effective than sestamibi scan in finding the parathyromatosis. In this case, ^99m^Tc-MIBI/SPECT failed to find the suspected parathyroid tissues in the neck, but US detected two lesions with a small volume located at the primary site. Previous studies showed that the suspected parathyroid lesions could be identified accurately by using CEUS (35). Indeed, in this case, two lesions, detected by US, presented with a vascularization pattern of parathyroid tissues, which can help diagnose parathyromatosis and prepare a surgery plan. According to our clinical observation, parathyromatosis usually presents as multiple and small-sized foci with surrounding tissue, and these foci would distribute widely in the primary surgery site. Treatment is the surgical removal of all foci. However, berry picking surgery would be a failure for complete excision; thus, an extensive surgery is required with resection of multiple parathyromatosis foci and clearance of the central neck compartment (36). In this case, the extensive surgery was performed, and no recurrence was present on the postoperative 10 months.

Even though TPTX + AT increases the risk of recurrence, it is still widely performed for SHPT patients subjected to surgery because the recurrent site can be easy to identify and remove them under local anesthesia (24). Several researchers thought that selection of the appropriate autograft tissue, the smallest parathyroid gland tissues without adenomatous hyperplasia in initial surgery, would be helpful for avoiding the recurrent SHPT (37). The choice between TPTX with or without AT remains controversial. Li et al. conducted a meta-analysis of 10 cohort studies and 1 randomized controlled trial involving 1,108 patients to compare the two surgical methods. The study found no significant differences in surgical complications, all-cause mortality, persistent hyperparathyroidism, or symptom improvement between the two methods. However, TPTX reduced the risk of recurrence and the need for a second surgery due to recurrence or persistent hyperparathyroidism (38). Liu et al. also conducted a meta-analysis and found that compared to TPTX + AT, the TPTX group had a lower recurrence rate (OR = 0.2, 95%CI: 0.11–0.38, *P* < 0.01), with no significant differences in complications (39). Hou et al. conducted a network meta-analysis of 26 studies including 5,063 patients and concluded that TPTX + AT was the most effective and least harmful surgical treatment for low calcium and recurrence rate (40). Li et al. compared 62 patients who underwent TPTX with 42 patients who underwent TPTX with forearm autotransplantation (FAT) and found that TPTX + FAT patients had significantly improved postoperative quality of life and lower recurrence rates compared to TPTX patients (41). In general, there is no single best surgical option, only a more suitable one. For patients who need to avoid a second surgery, including those with thyroid disease, multiple neck surgeries, known recurrent laryngeal nerve injury, and general anesthesia intolerance, TPTX + AT is preferred. For patients with longer life expectancy or those who are unlikely to receive kidney transplantation, TPTX has advantages in preventing recurrent refractory secondary hyperparathyroidism (42). In this case, the autograft tissue was chosen from the smallest one among the four resected glands in the initial surgery, and the recurrence of this case is not definitely caused by hyperplasia of the autograft tissues.

While PTX is a highly effective therapeutic option for SHPT, the issue of recurrence remains. Thus, it is of utmost importance to focus on strategies to decrease the incidence of recurrent SHPT after initial surgery. Currently, a consensus on the diagnostic and therapeutic guidelines for initial surgery has yet to be reached. Nevertheless, based on current experience, these guidelines should entail preoperative radiologic imaging, iPTH monitoring, necessary exploration, and possible tissue clearance. Previous studies showed that preoperative localization procedures are useful for complete resection (11, 43–48). Ultrasonography (USG), computed tomography (CT), and ^99m^Tc-sestamibi (^99m^TC-MIBI) scans are often performed to localize PTGs. Each technique has its own advantages and disadvantages in terms of sensitivity and accuracy. The combinations of various imaging modalities are encouraged to improve the accuracy of preoperative positioning, especially for SPGs and ectopic glands and the glands in the patients with recurrent SHPT (11, 29, 44, 46, 47). In this case, a functional gland in the mediastinum had been detected only by using ^99m^TC-MIBI/SPECT, and parathyromatosis foci had been found by means of CEUS. Moreover, intraoperative iPTH monitoring, an important tool, is widely used for preventing inadequate surgery in PTX (49, 50). Hiramitsu et al. reported that a 70% intraoperative iPTH drop from baseline 10 min after PTX is appropriate to determine sufficient PTG removal (49). If <70% intraoperative iPTH drop was observed, it is recommended to perform exploration and purging parathyroidectomy (PPTX). This involves a comprehensive resection of cervical fibro-fatty tissues, including the thymus tongue, that are enclosed by the thyroid cartilage, bilateral carotid sheath, and innominate artery (51).

In conclusion, postoperative recurrence of SHPT caused by SPGs and parathyromatosis should receive more attention. Multiple imaging modalities are valuable for precise preoperative localization of suspected parathyroid tissues and may help with subsequent treatment. An extensive surgery needs to be performed for patients with parathyromatosis that is located at the primary surgery site. Thoracoscopic surgery is a feasible and safe technique in the management of mediastinal parathyroid gland.

## Data Availability

The original contributions presented in the study are included in the article/Supplementary Material, further inquiries can be directed to the corresponding authors.

## References

[1] Hedgeman E Lipworth L Lowe K Saran R Do T Fryzek J. International burden of chronic kidney disease and secondary hyperparathyroidism: a systematic review of the literature and available data. Int J Nephrol. (2015) 2015:184321. 10.1155/2015/184321 25918645 PMC4396737

[2] Cohen-Solal M Funck-Brentano T Urena Torres P. Bone fragility in patients with chronic kidney disease. Endocr Connect. (2020) 9(4):R93–101. 10.1530/EC-20-0039 32168473 PMC7219138

[3] Kim D Pollock C. Epidemiology and burden of chronic kidney disease-associated pruritus. Clin Kidney J. (2021) 14(Suppl 3):i1–7. 10.1093/ckj/sfab142 34987777 PMC8702817

[4] Bozic M Diaz-Tocados JM Bermudez-Lopez M Forné C Martinez C Fernandez E Independent effects of secondary hyperparathyroidism and hyperphosphataemia on chronic kidney disease progression and cardiovascular events: an analysis from the NEFRONA cohort. Nephrol Dial Transplant. (2022) 37(4):663–72. 10.1093/ndt/gfab184 34021359

[5] Ogata H Kumasawa J Fukuma S Mizobuchi M Kinugasa E Fukagawa M The cardiothoracic ratio and all-cause and cardiovascular disease mortality in patients undergoing maintenance hemodialysis: results of the MBD-5D study. Clin Exp Nephrol. (2017) 21(5):797–806. 10.1007/s10157-017-1380-2 28508128 PMC5648748

[6] Steinl GK Kuo JH. Surgical management of secondary hyperparathyroidism. Kidney Int Rep. (2021) 6(2):254–64. 10.1016/j.ekir.2020.11.023 33615051 PMC7879113

[7] Filho WA van der Plas WY Brescia MDG Nascimento CP Jr. Goldenstein PT Neto LMM Quality of life after surgery in secondary hyperparathyroidism, comparing subtotal parathyroidectomy with total parathyroidectomy with immediate parathyroid autograft: prospective randomized trial. Surgery. (2018) 164(5):978–85. 10.1016/j.surg.2018.06.032 30082137

[8] Costa-Hong V Jorgetti V Gowdak LH Moyses RM Krieger EM De Lima JJ. Parathyroidectomy reduces cardiovascular events and mortality in renal hyperparathyroidism. Surgery. (2007) 142(5):699–703. 10.1016/j.surg.2007.06.015 17981190

[9] Chen L Wang K Yu S Lai L Zhang X Yuan J Long-term mortality after parathyroidectomy among chronic kidney disease patients with secondary hyperparathyroidism: a systematic review and meta-analysis. Ren Fail. (2016) 38(7):1050–8. 10.1080/0886022X.2016.1184924 27198474

[10] Richards ML Wormuth J Bingener J Sirinek K. Parathyroidectomy in secondary hyperparathyroidism: is there an optimal operative management? Surgery. (2006) 139(2):174–80. 10.1016/j.surg.2005.08.036 16455325

[11] Andrade JS Mangussi-Gomes JP Rocha LA Ohe MN Rosano M das Neves MC Localization of ectopic and supernumerary parathyroid glands in patients with secondary and tertiary hyperparathyroidism: surgical description and correlation with preoperative ultrasonography and Tc99m-sestamibi scintigraphy. Braz J Otorhinolaryngol. (2014) 80(1):29–34. 10.5935/1808-8694.20140008 24626889 PMC9443960

[12] Pattou FN Pellissier LC Noel C Wambergue F Huglo DG Proye CA. Supernumerary parathyroid glands: frequency and surgical significance in treatment of renal hyperparathyroidism. World J Surg. (2000) 24(11):1330–4. 10.1007/s002680010220 11038202

[13] Numano M Tominaga Y Uchida K Orihara A Tanaka Y Takagi H. Surgical significance of supernumerary parathyroid glands in renal hyperparathyroidism. World J Surg. (1998) 22(10):1098–102, discussion 1103. 10.1007/s002689900524 9747174

[14] Uno N Tominaga Y Matsuoka S Tsuzuki T Shimabukuro S Sato T Incidence of parathyroid glands located in thymus in patients with renal hyperparathyroidism. World J Surg. (2008) 32(11):2516–9. 10.1007/s00268-008-9739-x 18795242

[15] Hage MP Salti I El-Hajj Fuleihan G. Parathyromatosis: a rare yet problematic etiology of recurrent and persistent hyperparathyroidism. Metab Clin Exp. (2012) 61(6):762–75. 10.1016/j.metabol.2011.11.001 22221828

[16] Matsuoka S Tominaga Y Sato T Uno N Goto N Katayama A Recurrent renal hyperparathyroidism caused by parathyromatosis. World J Surg. (2007) 31(2):299–305. 10.1007/s00268-006-0391-z 17219279

[17] Rothmund M Wagner PK Schark C. Subtotal parathyroidectomy versus total parathyroidectomy and autotransplantation in secondary hyperparathyroidism: a randomized trial. World J Surg. (1991) 15(6):745–50. 10.1007/BF01665309 1767541

[18] Gasparri G Camandona M Abbona GC Papotti M Jeantet A Radice E Secondary and tertiary hyperparathyroidism: causes of recurrent disease after 446 parathyroidectomies. Ann Surg. (2001) 233(1):65–9. 10.1097/00000658-200101000-00011 11141227 PMC1421168

[19] Kievit AJ Tinnemans JG Idu MM Groothoff JW Surachno S Aronson DC. Outcome of total parathyroidectomy and autotransplantation as treatment of secondary and tertiary hyperparathyroidism in children and adults. World J Surg. (2010) 34(5):993–1000. 10.1007/s00268-010-0446-z 20145928 PMC2848726

[20] Schlosser K Bartsch DK Diener MK Seiler CM Bruckner T Nies C Total parathyroidectomy with routine thymectomy and autotransplantation versus total parathyroidectomy alone for secondary hyperparathyroidism: results of a nonconfirmatory multicenter prospective randomized controlled pilot trial. Ann Surg. (2016) 264(5):745–53. 10.1097/SLA.0000000000001875 27741007

[21] Abruzzo A Gioviale MC Damiano G Palumbo VD Buscemi S Lo Monte G Reoperation for persistent or recurrent secondary hyperparathyroidism. Acta Biomed. (2017) 88(3):325–8. 10.23750/abm.v88i3.4722 29083339 PMC6142843

[22] Steffen L Moffa G Muller PC Oertli D. Secondary hyperparathyroidism: recurrence after total parathyroidectomy with autotransplantation. Swiss Med Wkly. (2019) 149:w20160. 10.4414/smw.2019.20160 31800966

[23] Ishani A Liu J Wetmore JB Lowe KA Do T Bradbury BD Clinical outcomes after parathyroidectomy in a nationwide cohort of patients on hemodialysis. Clin J Am Soc Nephrol. (2015) 10(1):90–7. 10.2215/CJN.03520414 25516915 PMC4284409

[24] Tominaga Y Uchida K Haba T Katayama A Sato T Hibi Y More than 1,000 cases of total parathyroidectomy with forearm autograft for renal hyperparathyroidism. Am J Kidney Dis. (2001) 38(4 Suppl 1):S168–171. 10.1053/ajkd.2001.27432 11576947

[25] Cunningham J Locatelli F Rodriguez M. Secondary hyperparathyroidism: pathogenesis, disease progression, and therapeutic options. Clin J Am Soc Nephrol. (2011) 6:913–21. 10.2215/CJN.06040710 21454719

[26] Nakajima K Umino K Azuma Y Kosaka S Takano K Obara T Stimulating parathyroid cell proliferation and PTH release with phosphate in organ cultures obtained from patients with primary and secondary hyperparathyroidism for a prolonged period. J Bone Min Metab. (2009) 27(2):224–33. 10.1007/s00774-008-0032-8 19194773

[27] Wu Y Han W Li P Hu X Zhang Y. Impact of dexmedetomidine on secondary hyperparathyroidism recurrence in uremic patients who received parathyroidectomy with auto-transplantation: a retrospective propensity-matched study. Am J Transl Res. (2022) 14(9):6659–68. PMID: 36247273 36247273 PMC9556500

[28] Stracke S Keller F Steinbach G Henne-Bruns D Wuerl P. Long-term outcome after total parathyroidectomy for the management of secondary hyperparathyroidism. Nephron Clin Pract. (2009) 111(2):c102–9. 10.1159/000191200 19142022

[29] Hiramitsu T Tomosugi T Okada M Futamura K Tsujita M Goto N Pre-operative localisation of the parathyroid glands in secondary hyperparathyroidism: a retrospective cohort study. Sci Rep. (2019) 9(1):14634. 10.1038/s41598-019-51265-y 31602011 PMC6787184

[30] Nilubol N Beyer T Prinz RA Solorzano CC. Mediastinal hyperfunctioning parathyroids: incidence, evolving treatment, and outcome. Am J Surg. (2007) 194(1):53–6. 10.1016/j.amjsurg.2006.11.019 17560909

[31] Russell CF Edis AJ Scholz DA Sheedy PF van Heerden JA. Mediastinal parathyroid tumors: experience with 38 tumors requiring mediastinotomy for removal. Ann Surg. (1981) 193(6):805–9. 10.1097/00000658-198106000-00016 7247524 PMC1345178

[32] Cupisti K Dotzenrath C Simon D Roher HD Goretzki PE. Therapy of suspected intrathoracic parathyroid adenomas. Experiences using open transthoracic approach and video-assisted thoracoscopic surgery. Langenbecks Arch Surg. (2002) 386(7):488–93. 10.1007/s00423-001-0254-x 11819104

[33] Lu HI Chou FF Chi SY Huang SC. Thoracoscopic removal of hypertrophic mediastinal parathyroid glands in recurrent secondary hyperparathyroidism. World J Surg. (2015) 39(2):400–9. 10.1007/s00268-014-2797-3 25245433

[34] Randone B Costi R Scatton O Fulla Y Bertagna X Soubrane O Thoracoscopic removal of mediastinal parathyroid glands: a critical appraisal of an emerging technique. Ann Surg. (2010) 251(4):717–21. 10.1097/SLA.0b013e3181c1cfb0 19858697

[35] Pavlovics S Radzina M Niciporuka R Ratniece M Mikelsone M Tauvena E Contrast-enhanced ultrasound qualitative and quantitative characteristics of parathyroid gland lesions. Medicina. (2021) 58(1):2. 10.3390/medicina58010002 35056309 PMC8778856

[36] Achour I Charfi S Chaabouni MA Chakroun A Guermazi F Hammami B Parathyromatosis: an uncommon cause of recurrent hyperparathyroidism. Rev Med Interne. (2017) 38(1):61–4. 10.1016/j.revmed.2016.03.005 27083335

[37] Taieb D Hindie E Grassetto G Colletti PM Rubello D. Parathyroid scintigraphy: when, how, and why? A concise systematic review. Clin Nucl Med. (2012) 37(6):568–74. 10.1097/RLU.0b013e318251e408 22614188

[38] Li C Lv L Wang H Wang X Yu B Xu Y Total parathyroidectomy versus total parathyroidectomy with autotransplantation for secondary hyperparathyroidism: systematic review and meta-analysis. Ren Fail. (2017) 39(1):678–87. 10.1080/0886022X.2017.1363779 28853301 PMC6446159

[39] Liu ME Qiu NC Zha SL Du ZP Wang YF Wang Q To assess the effects of parathyroidectomy (TPTX versus TPTX+AT) for secondary hyperparathyroidism in chronic renal failure: a systematic review and meta-analysis. Int J Surg. (2017) 44:353–62. 10.1016/j.ijsu.2017.06.029 28634117

[40] Hou J Shan H Zhang Y Deng X Guo B Kang J Network meta-analysis of surgical treatment for secondary hyperparathyroidism. Am J Otolaryngol. (2020) 41(2):102370. 10.1016/j.amjoto.2019.102370 31889554

[41] Li JG Xiao ZS Hu XJ Li Y Zhang X Zhang SZ Total parathyroidectomy with forearm auto-transplantation improves the quality of life and reduces the recurrence of secondary hyperparathyroidism in chronic kidney disease patients. Medicine. (2017) 96(49):e9050. 10.1097/MD.0000000000009050 29245308 PMC5728923

[42] Lau WL Obi Y Kalantar-Zadeh K. Parathyroidectomy in the management of secondary hyperparathyroidism. Clin J Am Soc Nephrol. (2018) 13(6):952–61. 10.2215/CJN.10390917 29523679 PMC5989682

[43] Yin L Guo D Liu J Yan J. The role of ^99m^Tc-MIBI SPECT-CT in reoperation therapy of persistent hyperparathyroidism patients. Open Med. (2015) 10(1):462–7. 10.1515/med-2015-0064 PMC5368852 28352737

[44] Yuan LL Kan Y Ma DQ Yang JG. Combined application of ultrasound and SPECT/CT has incremental value in detecting parathyroid tissue in SHPT patients. Diagn Interv Imaging. (2016) 97(2):219–25. 10.1016/j.diii.2015.08.007 26432401

[45] Zeng M Liu W Zha X Tang S Liu J Yang G ^99m^Tc-MIBI SPECT/CT imaging had high sensitivity in accurate localization of parathyroids before parathyroidectomy for patients with secondary hyperparathyroidism. Ren Fail. (2019) 41(1):885–92. 10.1080/0886022X.2019.1662804 31537128 PMC6758704

[46] Zhang R Zhang Z Huang P Li Z Hu R Zhang J Diagnostic performance of ultrasonography, dual-phase ^99m^Tc-MIBI scintigraphy, early and delayed ^99m^Tc-MIBI SPECT/CT in preoperative parathyroid gland localization in secondary hyperparathyroidism. BMC Med Imaging. (2020) 20(1):91. 10.1186/s12880-020-00490-3 32746794 PMC7398336

[47] Li X Li J Li Y Wang H Yang J Mou S The role of preoperative ultrasound, contrast-enhanced ultrasound, and ^99m^Tc-MIBI scanning with single-photon emission computed tomography/x-ray computed tomography localization in refractory secondary hyperparathyroidism. Clin Hemorheol Microcirc. (2020) 75(1):35–46. 10.3233/CH-190723 31868660

[48] Jiang SQ Yang T Zou Q Xu L Ye T Kang YQ The role of ^99m^Tc-MIBI SPECT/CT in patients with secondary hyperparathyroidism: comparison with ^99m^Tc-MIBI planar scintigraphy and ultrasonography. BMC Med Imaging. (2020) 20(1):115. 10.1186/s12880-020-00517-9 33059621 PMC7565325

[49] Hiramitsu T Tominaga Y Okada M Yamamoto T Kobayashi T. A retrospective study of the impact of intraoperative intact parathyroid hormone monitoring during total parathyroidectomy for secondary hyperparathyroidism: STARD study. Medicine. (2015) 94(29):e1213. 10.1097/MD.0000000000001213 26200645 PMC4603015

[50] Zhang L Xing C Shen C Zeng M Yang G Mao H Diagnostic accuracy study of intraoperative and perioperative serum intact PTH level for successful parathyroidectomy in 501 secondary hyperparathyroidism patients. Sci Rep. (2016) 6:26841. 10.1038/srep26841 27231027 PMC4882599

[51] Shan CX Qiu NC Zha SL Liu ME Wang Q Zhu PP A novel surgical strategy for secondary hyperparathyroidism: purge parathyroidectomy. Int J Surg. (2017) 43:112–8. 10.1016/j.ijsu.2017.05.062 28578084

